# The Regulatory Role of FABP4 in Microbiome–Brain–Gut Communication Under High-Fat-Diet Conditions

**DOI:** 10.3390/ijms27052430

**Published:** 2026-03-06

**Authors:** Katarzyna Smolińska, Ewa Tomaszewska, Monika Hułas-Stasiak, Siemowit Muszyński, Aleksandra Szopa, Anna Serefko, Piotr Dobrowolski

**Affiliations:** 1 Chronic Wounds Laboratory, Medical University of Lublin, Chodźki St. 7, 20-093 Lublin, Poland; katarzyna.smolinska@umlub.edu.pl; 2 Department of Animal Physiology, University of Life Sciences in Lublin, 20-950 Lublin, Poland; ewarst@interia.pl; 3 Department of Functional Anatomy and Cytobiology, Maria Curie Sklodowska University, Akademicka St. 19, 20-033 Lublin, Poland; monika.hulas-stasiak@mail.umcs.pl; 4 Department of Biophysics, University of Life Sciences in Lublin, 20-950 Lublin, Poland; siemowit.muszynski@up.lublin.pl; 5 Department of Clinical Pharmacy and Pharmaceutical Care, Medical University of Lublin, Chodźki St. 1, 20-093 Lublin, Poland; aleksandra.szopa@umlub.edu.pl (A.S.); anna.serefko@umlub.edu.pl (A.S.)

**Keywords:** fatty acid-binding protein 4, FABP4, high-fat diet, gut microbiota, gut–brain axis, intestinal barrier, neuroinflammation, metabolic inflammation

## Abstract

High-fat diets (HFDs) are major environmental factors influencing metabolic homeostasis, immune regulation, and brain function, largely through their effects on gut microbiota and intestinal barrier integrity. Disruption of the microbiome–brain–gut axis has been increasingly implicated in systemic and neuroinflammatory processes; however, the molecular mediators that integrate dietary lipid signals with microbial and host responses remain incompletely defined. This review synthesizes the current evidence on the role of fatty acid-binding protein 4 (FABP4) as an integrative node linking HFD-induced gut dysbiosis to systemic and central inflammatory signaling. We critically evaluated experimental and translational studies addressing HFD-driven alterations in gut microbiota composition, intestinal barrier function, and inflammatory pathways, with particular emphasis on FABP4-mediated mechanisms across epithelial, immune, and neural compartments. The available data indicate that FABP4 responds to dietary and microbiome-derived cues and contributes to coordinated metabolic and inflammatory responses, affecting both peripheral tissues and the central nervous system. These findings support a model in which FABP4 participates in diet-driven feedback loops that amplify gut barrier dysfunction, immune activation, and neuroinflammation. In conclusion, FABP4 emerges as a central molecular mediator within the microbiome–brain–gut axis under HFD conditions, highlighting its potential relevance in understanding the pathophysiology of metabolic and neuroinflammatory disorders and guiding future integrative research strategies.

## 1. Introduction

Diet plays a crucial role in shaping human physiology and influencing metabolism, immunity, and brain function [1]. High-fat diets (HFDs), which cause obesity, impact the gut microbiome and gastrointestinal tract, leading to changes in microbial composition and gut barrier integrity [2,3]. These alterations affect peripheral immune responses and central nervous system (CNS) function via neuroimmune pathways [4,5]. Signals from the microbiome, along with dietary lipids, converge on proteins such as fatty acid-binding protein 4 (FABP4), which is expressed in adipocytes, macrophages, endothelial cells, and Paneth cells [6,7]. FABP4 mediates microbial and inflammatory signals, with microbiota-derived fatty acids modulating its expression [8,9]. FABP4 orchestrates metabolic responses in diverse tissues [8,10].

The brain–gut axis is a bidirectional network that integrates neural, endocrine, immune, and microbial signals [11]. The gastrointestinal tract sends sensory and microbial inputs to the CNS, whereas neural pathways regulate gut function [12]. Key components include the enteric nervous system, vagal afferents, enteroendocrine cells, immune cells, and gut microbiota, which produce metabolites that signal through their receptors [13,14]. This axis maintains homeostasis and modulates cognitive processes [11], but its disruption can trigger maladaptive responses that reach the brain through various pathways [15].

There is growing evidence that disturbances in the microbiota contribute to the development of chronic diseases [16]. Dysbiosis has been associated with obesity, metabolic syndrome, inflammatory bowel disease, depression, and neurodegenerative disorders [17,18,19,20,21,22].

Western diets rich in saturated fats shift the microbiome towards pro-inflammatory profiles, increasing endotoxin exposure and compromising gut barrier integrity [23,24]. HFDs induce changes in blood–brain barrier (BBB) permeability even before weight gain [25,26], eventually leading to neuroinflammation and cognitive decline [27,28]. The role of FABP4 in neuroinflammatory signaling adds complexity to our understanding of the brain–gut axis [29].

FABP4 plays a role in both systemic and CNS effects. In adipocytes, it influences lipolysis, inflammatory signaling, and adipokine secretion [30]. Under HFD conditions, the circulating levels of FABP4 increase due to its expression in adipose tissue and immune cells [31,32]. Metabolites derived from the microbiome can modulate FABP4 expression, linking diet, dysbiosis, and neuroinflammation [8,30].

Recent research has broadened our understanding of FABP4 function, highlighting its role in epithelial and neuroimmune regulation [33]. This positions FABP4 as a pivotal molecular switch for maintaining epithelial–microbial homeostasis. In the CNS, microglial FABP4 is implicated in diet-induced neuroinflammation [34,35,36]. FABP4 facilitates oxidative stress and inflammatory pathways [34], thereby linking peripheral inflammation to microglial activation and cognitive decline [34,36].

Emerging studies suggest that FABP4 responds to dietary fat intake, inflammatory stimuli, and microbiome-derived signals, positioning it as a potential mediator that connects gut dysbiosis to systemic and neuroinflammatory outcomes. HFD-induced alterations in gut barrier function and endotoxin translocation are increasingly recognized as early events linking dietary composition to systemic inflammation, providing a biological context in which FABP4-mediated signaling may be particularly relevant.

In this review, we synthesize the current evidence on the regulatory role of FABP4 in the microbiome–brain–gut axis under HFD conditions. We focused on how HFD-induced changes in the gut microbiota and microbial metabolites intersect with FABP4 signaling and how this interaction contributes to intestinal barrier dysfunction, systemic inflammation, and neuroinflammatory processes. By framing FABP4 as a signaling integrator in diet–microbiome–host communication, we aim to highlight its relevance as a biomarker and potential therapeutic target for metabolic and neuroinflammatory disorders.

The literature selection strategy and data synthesis procedures are described in detail in Section 8.

## 2. HFD-Induced Alterations of the Gut Microbiota

### 2.1. Changes in the Microbial Composition and Diversity

HFDs induce reproducible alterations in the gut microbiota composition and intestinal barrier function, forming a mechanistic link between dietary fat intake and systemic inflammation. Even without caloric excess, exposure to HFD diminishes microbial diversity and reconfigures community structure towards pro-inflammatory states, altering host–microbe interactions at the intestinal surface [3,5,37].

It is important to recognize that the biological effects of HFDs strongly depend on the diet composition, including the total fat content, fatty acid profile, carbohydrate source, and fiber levels. Experimental HFD models typically provide 45–60% of the total caloric intake from fat, often derived from lard or milk fat, whereas standard control diets contain approximately 10–15% fat. Variations in fiber content and refined carbohydrate composition may independently influence microbial diversity, short-chain fatty acid production and inflammatory tone. In addition, exposure duration, host genetic background, and sex significantly modulate the microbiota composition and metabolic outcomes. Therefore, the interpretation of HFD-induced dysbiosis should consider these contextual factors [2,3,38].

HFD-induced dysbiosis decreases alpha diversity and shifts the dominant bacterial phyla [5,39]. Studies have indicated an increased Firmicutes-to-Bacteroidetes (F/B) ratio, although this metric does not fully capture the functional changes in the microbiome [37,38]. HFD depletes fiber-fermenting and short-chain fatty acid (SCFA)-producing bacteria while enriching bile- and lipid-tolerant taxa, including members of Enterobacteriaceae [39,40,41]. These changes, influenced by diet composition, exposure duration, host genotype, and sex, result in reduced SCFA availability and increased luminal endotoxin loads [37,39]. HFD may also encourage the expansion of fungal and archaeal communities, further affecting the inflammatory tone and epithelial stress responses [42].

### 2.2. Microbial Metabolites in Host Metabolic and Inflammatory Signaling

The functional consequences of dysbiosis are evident at the level of the intestinal barrier. SCFAs, particularly butyrate, serve as the primary energy source for colonocytes and support tight junction assembly, epithelial differentiation, and immune tolerance [43]. The loss of SCFA-producing bacteria under HFD conditions compromises epithelial resilience and leads to barrier dysfunction. The enrichment of Gram-negative bacteria increases lipopolysaccharide (LPS) levels in the gut lumen, creating conditions for metabolic endotoxemia [38].

HFD disrupts the integrity of tight junctions through multiple mechanisms. Reduced expression and altered localization of junctional proteins, including occludin, claudins, and zonula occludens-1 (ZO-1), occur following HFD feeding [38,44,45]. Pro-inflammatory cytokines, such as tumor necrosis factor α (TNF-α) and interleukin-1 β (IL-1β), activate myosin light chain kinase (MLCK), causing phosphorylation of myosin light chains, actin–myosin contraction, and increased paracellular permeability [46,47]. Cytokine-induced miRNAs suppress tight junction gene expression, further increasing epithelial leakiness [47]. These processes undermine the physical barrier between luminal microbes and the host.

Altered bile acid metabolism links HFD to epithelial damage. The increase in primary bile acids and their microbial conversion into hydrophobic secondary bile acids, such as deoxycholic acid, leads to oxidative stress, mitochondrial dysfunction, and apoptosis in epithelial cells [38]. These hydrophobic bile acids destabilize tight junctions and hinder epithelial renewal, exacerbating the breakdown of the barrier [44]. Excessive lipid metabolism in enterocytes further contributes to oxidative stress and inflammatory signaling, increasing epithelial vulnerability under HFD conditions [38]. In addition, HFD impairs mucus-mediated protection. Suppression of peroxisome proliferator-activated receptor γ (PPARγ) signaling in goblet cells reduces mucin 2 (MUC2) synthesis and secretion, thinning the mucus layer and increasing bacterial–epithelial contact [38,48]. Reduced production of antimicrobial peptides compromises host–microbe segregation, facilitating bacterial translocation and immune activation [38].

**Figure 1:**
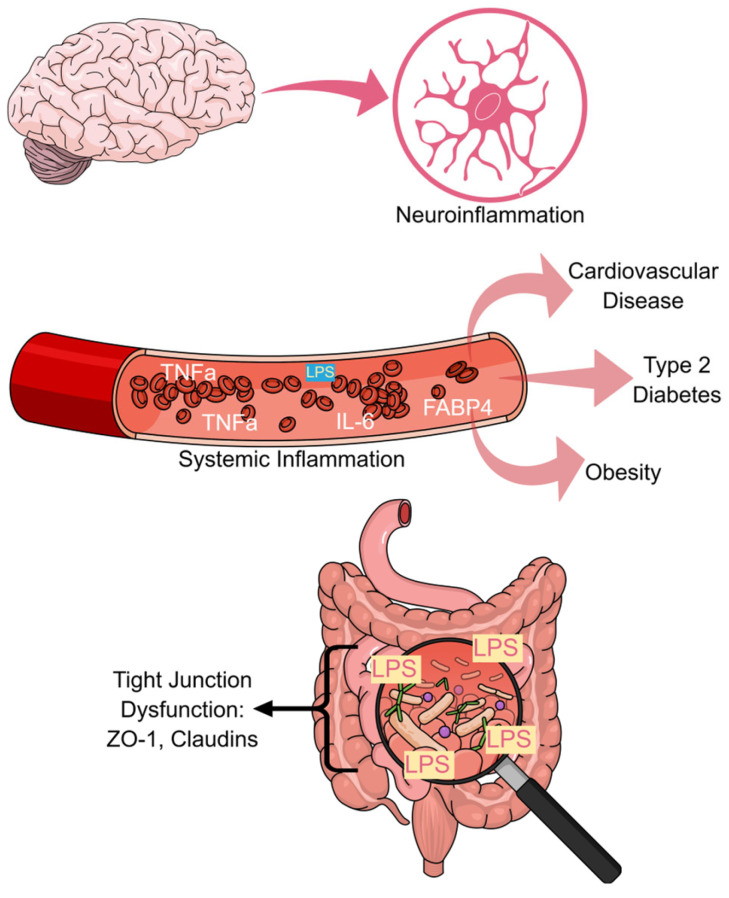
High-fat diet-induced gut dysbiosis and intestinal barrier dysfunction as drivers of systemic inflammation. High-fat diets (HFDs) alter the gut microbiota composition, reducing microbial diversity and enriching pro-inflammatory taxa, including Gram-negative bacteria. Dysbiosis leads to decreased production of protective microbial metabolites, such as short-chain fatty acids (SCFAs), and increased levels of luminal lipopolysaccharide (LPS). These changes impair intestinal barrier integrity by disrupting tight junction proteins (e.g., ZO-1, claudins, and occludin), epithelial apoptosis, and reducing mucus and antimicrobial peptide production. Barrier dysfunction facilitates the translocation of microbial products into the lamina propria and systemic circulation, resulting in metabolic endotoxemia and chronic low-grade inflammation, which contribute to metabolic and inflammatory disorders. This illustration was created using Mind the Graph (https://mindthegraph.com, accessed on 26–28 January 2026).

As a result of these combined defects, microbial products, including LPS, access the lamina propria and systemic circulation, leading to metabolic endotoxemia and chronic, low-grade inflammation [37,38]. Circulating LPS activates Toll-like receptor 4 (TLR4) signaling in immune cells, adipose tissue, and metabolic organs, driving nuclear factor kappa-light-chain-enhancer of activated B cells (NF-κB)-dependent cytokine production and inflammatory feedback loops [38]. These processes are strongly associated with insulin resistance, adipose inflammation, hepatic steatosis, and endothelial dysfunction in HFD-fed animal models [40,49]. Intestinal barrier dysfunction precedes and predicts systemic inflammatory responses. Studies have indicated that restoring gut barrier integrity through fiber supplementation, prebiotics, or microbiota transplantation reduces endotoxemia and improves metabolic parameters, even with a continued high dietary fat [50,51]. These findings underscore the role of the intestinal barrier as a gatekeeper in regulating the systemic effects of diet-induced dysbiosis. These interconnected mechanisms are illustrated in Figure 1.

## 3. HFD-Driven Microbiome-FABP4 Crosstalk: Evidence from Animal and Human Studies

The processes of HFD-induced gut dysbiosis and intestinal barrier dysfunction are interconnected, transforming excess dietary lipids into systemic inflammatory signals. By altering microbial communities, depleting protective metabolites, and weakening epithelial defenses, HFD fosters an environment conducive to metabolic endotoxemia and chronic inflammation, affecting both peripheral tissues and the CNS. HFD-induced alterations in gut microbiota composition are crucial upstream factors influencing FABP4 regulation in various tissues. Evidence from animal models, in vitro systems, and human studies increasingly suggests that microbiome-derived metabolites, such as SCFAs, bile acids, and oxidized lipid species, serve as key modulators of FABP4 expression, secretion, and activity. These microbial signals integrate dietary lipid exposure with host metabolic and inflammatory pathways, positioning FABP4 at the intersection of gut dysbiosis and systemic response. They influence FABP4 expression and function through molecular mechanisms such as receptor-mediated signaling, neuroendocrine pathways, nuclear receptor crosstalk, epigenetic regulation, and direct ligand binding. Notably, the regulatory effects of the microbiome on FABP4 are context-dependent and influenced by both dietary composition and the specificity of host tissues [8,9,52,53].

Zebrafish and rodent models are valuable tools for investigating the effects of diet and microbiota on metabolism and behavior. Zebrafish facilitate rapid in vivo experiments with fluorescent imaging [54,55], whereas mice enable biochemical and behavioral analyses following genetic and microbiota manipulations [56]. Transparent zebrafish larvae are an exceptional model for in vivo intestinal observation. Their optical clarity allows the tracking of bacterial colonization by introducing fluorescently labeled bacteria into the aquarium [54,55]. Russo et al. (2015) demonstrated that probiotic bacteria containing *mCherry* genes colonize the larvae’s intestines, which can be observed through fluorescence microscopy [55]. Genetic modifications facilitate the study of specific host genes, such as transgenic lines in which the *FABP4* gene promoter drives enhanced green fluorescent protein expression to visualize adipocytes [57]. These systems enable real-time localization of metabolic markers. Zebrafish can be fed various dietary components or bacterial strains to assess their effects on the microbiome and metabolism [58]. While zebrafish models provide valuable tools for visualizing adipocyte dynamics and microbiota–host interactions in vivo, most mechanistic evidence linking FABP4 to microbiome–brain–gut signaling pathways is derived from rodent models and cellular studies. Therefore, zebrafish findings should be interpreted primarily as supportive and exploratory, rather than as definitive mechanistic evidence.

Rodent models, particularly mice, such as the C57BL/6 strain, are invaluable for studying the metabolic, neurobehavioral, and immunological changes following HFD and microbiota interventions [59]. HFD in C57BL/6 mice leads to obesity, inflammation, insulin resistance, and behavioral disorders, such as anxiety and depression. HFD elevates pro-inflammatory cytokines in the brain, including TNF-α and IL-1β, which can be reduced by antibiotics [59]. Modifying the microbiome using antibiotics, probiotics, and microbiota transplants can alter disease progression. Notably, fecal transplants from obese animals induce cognitive deficits in recipients [60]. In genetic models, FABP4 knockout mice exhibit pro-inflammatory-related inflammation and insulin resistance, even when fed an HFD [56]. These models are crucial for studying the role of FABP4 in metabolic and immune signaling pathways.

Observations in the population indicate that obesity and metabolic syndrome are linked to dysbiosis and intestinal inflammation [61]. Elevated FABP4 levels are associated with metabolic parameters and inflammatory markers [62]. As a potential mechanism, it has been suggested that HFD may increase FABP4 levels in intestinal cells, which inhibits defensins and promotes dysbiosis. These changes in the microbiome can worsen inflammation and disrupt the gut–brain axis [33]. However, no study has explored the effects of microbiota modification on FABP4 and brain function. Such research would be instrumental in confirming correlations and understanding causality within the gut–brain system.

Given the emerging evidence positioning FABP4 as a mediator of microbiome-driven metabolic and inflammatory signaling, a closer examination of its molecular structure and regulatory mechanisms is warranted to better understand the execution of these interactions at the cellular level.

## 4. FABP4: Molecular Functions and Regulatory Mechanisms

FABP4 belongs to the fatty acid-binding protein family and possesses structural and biochemical characteristics that support selective interactions with hydrophobic lipids. It exhibits a conserved FABP fold, forming an internal binding cavity that accommodates long-chain fatty acids and related lipid species, thereby functioning as a high-affinity intracellular lipid chaperone. Binding is driven by hydrophobic interactions within this cavity and is reversible, enabling FABP4 to shuttle lipid ligands between cellular membranes and enzymes involved in metabolic and signaling pathways. FABP4 functions as an intracellular lipid chaperone and extracellular adipokine that modulates lipid metabolism, signaling cascades, and intercellular communication. These dual roles allow FABP4 to influence lipid fluxes inside cells and to act hormonally on distant tissues, linking adipose tissue status to systemic metabolic responses [31,63].

### 4.1. FABP4 Structure, Expression, and Lipid-Binding Properties

FABP4, a lipid chaperone and inflammatory mediator, functions locally in the intestinal epithelium and systemically. It is a key molecular integrator that translates dietary and microbiome-derived lipid signals into coordinated metabolic and inflammatory responses. Rather than acting as a passive lipid chaperone, FABP4 functions as an active regulatory interface that connects epithelial defense mechanisms, immune activation, and neuroinflammatory signaling [29].

Structurally, FABP4 is a ~14–15 kDa cytosolic protein composed of a 10-stranded antiparallel β-barrel capped by two short α-helices that form a helix–turn–helix motif. The β-barrel creates an internal hydrophobic cavity that accommodates long-chain fatty acids and other lipophilic ligands. Key positively charged residues within the binding pocket stabilize the carboxylate group of fatty acids, enabling high-affinity, yet reversible ligand interactions. Conformational flexibility in the portal region facilitates ligand exchange and interaction with partner proteins, allowing FABP4 to coordinate intracellular lipid trafficking and signaling [63].

The studies summarized herein indicate that FABP4 operates at multiple anatomical and functional levels. In the intestine, FABP4 influences epithelial–microbial interactions and barrier function; systemically, it circulates as an adipokine that amplifies inflammatory responses; and in the CNS, FABP4 contributes to microglial immunometabolism and diet-induced neuroinflammation. These interconnected roles position FABP4 at the intersection of diet, microbiota, and host inflammatory tone [64].

The central role of FABP4 in the microbiome–brain–gut axis suggests important translational implications. Circulating FABP4 levels may serve as a sensitive biomarker for combined disturbances in lipid metabolism, microbiota-derived signaling, and inflammatory status. Importantly, FABP4 integrates information on dietary composition and microbial metabolism, offering a broader physiological readout than markers focused on single organs [29].

Therapeutically, strategies aimed at modulating FABP4 activity could complement dietary and microbiome-based interventions to prevent obesity. Pharmacological inhibition of FABP4, activation of protective nuclear receptor pathways, and dietary approaches that restore beneficial microbial metabolites may collectively attenuate systemic and neuroinflammatory outcomes. Rather than replacing lifestyle interventions, FABP4-targeted approaches may enhance their effectiveness by disrupting maladaptive inflammatory feedback loops [65].

### 4.2. Regulation of FABP4 by Microbial Metabolites and Dietary Lipids

Recent experimental studies have identified microbiome-derived metabolites, particularly SCFAs, bile acids, and oxidized lipids, as key regulators of FABP4 (Table 1). SCFAs have emerged as crucial modulators of FABP4 regulation. In both mice and humans, propionate elevates circulating FABP4 and glucagon levels through sympathetic activation and endocrine signaling rather than by directly acting on adipocytes [8]. This neuroendocrine pathway illustrates how gut-derived metabolites influence systemic FABP4 release and metabolic responses. In contrast, acetate has cell type-specific effects on FABP4 expression within the immune system. Specifically, gut-derived acetate suppresses FABP4 expression in neutrophils via free fatty acid receptor 2 (FFAR2) activation, which in turn alters apoptotic responses and endoplasmic reticulum (ER) stress pathways in sepsis and acute lung injury models [8,9]. Additionally, SCFAs may regulate FABP4 transcription by acting as histone deacetylase inhibitors, suggesting an epigenetic control mechanism in both adipose and immune cells [66].

Bile acids are a significant class of microbiome-modulated FABP4 regulators. Increased secondary bile acids, driven by diet and microbiota, are associated with elevated FABP4 mRNA expression in the intestinal and adipose tissues. This process is mediated through nuclear receptor signaling pathways involving the farnesoid X receptor (FXR) and PPARs [53]. Computational docking and structural modeling suggest that specific bile acids, such as lithocholic acid and deoxycholic acid, may directly bind to FABP4, indicating that bile acids influence FABP4 activity through receptor-dependent and ligand-mediated mechanisms [69]. These regulatory modes position FABP4 as a molecular interface that integrates bile acid signaling with lipid trafficking and inflammation. Oxidized and nitrated lipids constitute a third class of microbiome- and inflammation-associated metabolites that regulate FABP4. Nitro fatty acids (NO_2_-FAs) and oxidized fatty acids, such as hydroxyoctadecadienoic acids (HODEs), bind FABP4 with high affinity, activate PPARγ-dependent transcription, and induce FABP4 expression [67,68]. This interaction creates an amplification loop in which FABP4 acts as a lipid chaperone, enhancing PPARγ-driven gene expression. FABP4 influences the stability of PPARγ by facilitating its ubiquitination and subsequent degradation, thereby creating a regulated mechanism that balances transcriptional activation with protein turnover [70].

Mechanistically, these metabolite classes converge on FABP4 regulation via interconnected signaling pathways. SCFA-induced G protein-coupled receptor (GPR) activation, sympathetic outflow, and endocrine signaling work together to coordinate the release of FABP4 from adipose tissue and immune cells [8,9,66]. Nuclear receptor crosstalk integrates these signals, with oxidized lipids acting as partial PPARγ agonists and FABP4 as a binding partner to enhance transcriptional activity [67]. Direct ligand binding by electrophilic fatty acids and bile acids positions FABP4 as a central hub in intracellular lipid signaling, allowing it to sense changes in microbial and dietary lipids and translate these changes into altered nuclear receptor signaling and inflammatory responses [67,69]. These processes are modulated by cellular stress pathways, particularly ER stress, which connects acetate–FFAR2 signaling to neutrophil survival and inflammatory outcomes in acute disease models [9].

Translational studies have highlighted the physiological significance of metabolite-FABP4 interactions. Randomized experiments have revealed that oral propionate increases the levels of norepinephrine, glucagon, and FABP4, resulting in temporary insulin resistance; these effects diminish in the absence of FABP4 or glucagon receptor signaling [8]. In sepsis and acute respiratory distress syndrome models, disruption of the acetate–FFAR2–FABP4 pathway affects ER stress responses, neutrophil apoptosis, and the severity of lung injury, demonstrating the context-dependent role of FABP4 regulation [9]. Studies on NO_2_-FAs have identified FABP4 as a participant in anti-inflammatory and antioxidant lipid signaling, reinforcing PPARγ-driven transcriptional programs [67]. Dietary intervention studies support microbiota-driven modulation of FABP4 through bile acid–FXR/PPAR signaling, whereas computational analyses bolster evidence for FABP4–bile acid interactions [53,69]. Emerging data extend these pathways beyond metabolic diseases, with sodium propionate regulating FABP4-linked signaling in ferroptosis and atopic dermatitis [71]. These findings indicate that microbiome-derived metabolites regulate FABP4 through receptor-mediated, transcriptional, epigenetic, and ligand-binding pathways. Collectively, these pathways place FABP4 at the interface between dietary lipid exposure, microbial metabolism and host inflammatory signaling.

## 5. FABP4 at the Intestinal Interface

### 5.1. Paneth Cell FABP4 and Epithelial Antimicrobial Defense

The intestinal epithelium is one of the body’s most crucial barriers, acting as an interface where nutrients, microbial signals, and dietary metabolites engage with host physiology. This tissue is responsible for absorbing essential nutrients while maintaining a selective barrier against pathogens and immune activation. A notable feature of the intestine is its specialized epithelial cells, each performing distinct yet complementary roles. Among these, Paneth cells occupy a unique position at the base of the intestinal crypts of the Lieberkühn. Traditionally, Paneth cells have been recognized for their role in innate defense, secreting antimicrobial peptides such as α-defensins, lysozyme, and regenerating islet-derived protein 3 γ [72]. These molecules are vital for preserving gut barrier integrity, shaping the microbial ecosystem, and protecting against pathogens [73,74]. A reduction in these molecules disrupts the ecological balance of the gut and has been associated with inflammatory bowel disease, obesity, and metabolic syndrome. Recent discoveries have broadened the functional repertoire of Paneth cells, revealing their involvement in metabolic regulation through factors previously thought to originate solely from the adipose tissue [7,33].

Dietary inputs play a crucial role in maintaining intestinal homeostasis, and HFDs are associated with impaired defensin expression and dysbiosis. Although this connection was previously recognized, the molecular pathways linking dietary fat to Paneth cell dysfunction were not well understood until recently [33,75]. Su et al. (2015) discovered that Paneth cells produce metabolic factors such as FABP4, adipsin, and adiponectin, challenging the notion that these proteins are exclusively derived from adipocytes or macrophages [7]. This study demonstrated that these factors are regulated by gut microorganisms, particularly *Lactobacillus species*, highlighting the role of intestinal epithelial cells as mediators of microbial and dietary signals that influence systemic metabolism. As a molecule derived from Paneth cells, FABP4 serves as a link between diet, microbiome, innate immunity, and host metabolism [7].

FABP4 integrates microbial and dietary signals within the gut epithelium, influencing systemic lipid and glucose homeostasis [7,33]. The ablation of Paneth cells induced by dithizone led to reduced levels of FABP4, adipsin, and adiponectin in the intestine and circulation, indicating that their production relies on intestinal epithelial sources. This ablation disrupts systemic lipid and glucose metabolism, suggesting that Paneth cell-derived proteins connect intestinal and peripheral metabolic regulation [7]. In germ-free mice, the expression of these proteins was nearly absent but was restored through fecal transplantation from wild-type mice [7]. Among the commensals, *Lactobacillus NK6* emerged as a potent inducer, elevating the intestinal and serum levels of these proteins and enhancing lipid and glucose metabolism. This effect was specific to Paneth cells, with no changes observed in the production of adipose tissue or macrophages [7].

*Lactobacillus NK6* influences Paneth cells through NF-κB activation rather than TLR2 signaling, which is typically associated with Gram-positive bacterial recognition [7]. Exposure to *Lactobacillus NK6* led to the phosphorylation of NF-κB p65 and activation of MAPK pathways, while also triggering the ubiquitination of TNF receptor-associated factor (TRAF) 2 and 6, specifically the K63-linked modification of TRAF6. In mice deficient in NF-κB, *Lactobacillus* failed to induce FABP4, adipsin, and adiponectin. These findings defined a microbiota–Paneth cell–systemic metabolism axis in which microbes regulate host metabolic signals through epithelial signaling [7]. Colon biopsies from patients with ulcerative colitis and Crohn’s disease showed an increase in Paneth cells and stronger immunostaining for FABP4, adipsin, and adiponectin than in healthy controls [7,76,77]. Su et al. (2022) identified FABP4 as a suppressor of antimicrobial peptide production [33]. FABP4 downregulates defensin expression by promoting the degradation of PPARγ, which drives the transcription of defensin genes. In cells with sufficient FABP4, it binds to phosphorylated PPARγ and triggers its proteasomal degradation, thereby reducing defensin expression. Mice lacking epithelial FABP4 (*Fabp4^fl/fl^; Villin^CreT^*) exhibited higher levels of defensins and resistance to *Salmonella* and *Escherichia coli* infections than their wild-type counterparts. Activation of PPARγ by pioglitazone restored defensin production, confirming the involvement of the FABP4–PPARγ axis [33].

High-fat feeding increased FABP4 levels in Paneth cells, decreased PPARγ expression, and suppressed defensin levels, thereby compromising host–microbe segregation. This results in dysbiosis, characterized by a higher F/B ratio, which is associated with obesity [33]. Palmitic acid replicated these changes via GPR40 activation. Mice deficient in FABP4 maintained defensin expression, microbial balance, and barrier integrity, establishing FABP4 as a mediator of HFD-induced defensin suppression and dysbiosis [33]. Human colonic Paneth-like cells (CD24^+^) and intestinal organoids expressed FABP4, and its silencing increased defensin levels and bactericidal activity, whereas overexpression or exposure to palmitic acid suppressed these effects, demonstrating FABP4’s evolutionary conservation in intestinal antimicrobial defense. FABP4 plays a dual role: microbiota regulate FABP4, adipsin, and adiponectin production via NF-κB signaling, affecting systemic metabolism, whereas dietary fats induce FABP4, suppressing defensin expression through PPARγ degradation and reshaping the gut microbiota. FABP4 operates within a bidirectional regulatory framework that integrates microbial and dietary signals, thereby affecting host physiology and microbial ecology [33].

Importantly, this relationship should not be interpreted as a strictly linear cause-and-effect sequence. Microbiota-derived signals may induce FABP4 expression in Paneth cells via NF-κB-dependent pathways, while elevated epithelial FABP4, particularly under high-fat-diet conditions, suppresses defensin production through PPARγ degradation and secondarily alters microbial composition. Thus, rather than representing a contradiction, these findings support the existence of a context-dependent feedback loop in which the microbial communities and FABP4 activity dynamically influence each other.

### 5.2. FABP4-Mediated Intestinal Barrier Dysfunction and Endotoxemia

FABP4 is involved in the regulation of intestinal barrier function and permeability. Intestinal barrier dysfunction is a characteristic of both gastrointestinal and systemic diseases, such as inflammatory bowel disease, metabolic syndrome, and sepsis [78,79]. The epithelial barrier protects against luminal antigens and pathogens while permitting nutrient absorption. When this barrier is compromised, it leads to increased intestinal permeability, bacterial translocation, and systemic inflammation [79]. In a healthy gut, PPARγ enhances the expression of anti-inflammatory genes, stabilizes tight junction proteins such as occludin, claudins, and ZO-1, and promotes barrier integrity [80,81]. HFD feeding, endotoxemia, or cytokine stimulation upregulate FABP4 in epithelial cells and macrophages. Elevated FABP4 levels sequester lipid ligands that activate PPARγ, thereby reducing its protective activity. Mechanistically, FABP4 binds to long-chain fatty acids and oxidized lipid species that serve as endogenous PPARγ ligands, thereby limiting their nuclear availability and attenuating PPARγ-dependent transcription of anti-inflammatory and barrier-protective genes. Reduced PPARγ activity permits enhanced NF-κB nuclear translocation and transcription of proinflammatory cytokines, establishing a reciprocal regulatory relationship between these pathways. Upregulation of FABP4 also enhances NF-κB activation, driving the expression of TNF-α, IL-1β, and interleukin 6 (IL-6). These cytokines disrupt tight junctions and increase permeability, creating a cascade in which FABP4 suppresses PPARγ, enhances NF-κB activation, and amplifies inflammation. This loop undermines epithelial barrier integrity, allowing bacterial product translocation into the circulation, fueling inflammation and FABP4 expression [82,83,84].

Evidence from non-intestinal cells supports this hypothesis. In IL-1β-stimulated chondrocytes, FABP4 knockdown reduced inflammation by restoring PPARγ activity and inhibiting NF-κB activation, whereas PPARγ inhibition reversed this effect. In endothelial cells exposed to LPS or oxidized low-density lipoprotein, FABP4 expression mirrored NF-κB activation, and FABP4 inhibition restored PPARγ activity while reducing inflammation. In cardiomyocytes, silencing FABP4 protected against LPS-induced hypertrophy by suppressing TLR4 and NF-κB signaling. Although much of the direct mechanistic evidence for the FABP4–PPARγ–NF-κB axis derives from non-intestinal cell types, including chondrocytes, endothelial cells, and cardiomyocytes, these studies provide important mechanistic insights into the conserved lipid–inflammatory signaling pathways. In the intestinal context, available data indicate that NF-κB activation promotes barrier dysfunction, whereas PPARγ agonists exert protective effects against colitis and injuries to the epithelium. While extrapolation across tissues should be interpreted with caution, the convergence of these signaling pathways supports the plausibility of a similar regulatory framework operating in intestinal epithelial and immune cells [64,85,86,87]. Collectively, these findings suggest that FABP4 may contribute to the regulation of barrier dysfunction through interactions with PPARγ and NF-κB signaling pathways and may represent a potential biomarker and therapeutic target in conditions associated with intestinal barrier impairment.

The broader network regulating FABP4 expression adds additional complexity. Song et al. (2021) demonstrated that matrix metalloproteinase-12 (MMP-12), a metalloproteinase derived from macrophages, functions as a non-classical transcriptional regulator of FABP4 in the intestine [82]. In response to HFD or free fatty acids, MMP-12 translocates to the nucleus and binds to the FABP4 promoter, facilitating transcription through histone acetylation [82]. This process enhances lipid absorption and pro-inflammatory signaling while diminishing tight junction proteins, thereby linking metabolic stress to barrier dysfunction. Studies on low-birth-weight piglets have revealed that early life programming alters FABP4 expression, disrupting lipid metabolism and the integrity of tight junctions. These findings underscore FABP4’s role as a crucial effector that connects diet, developmental cues, and barrier physiology [82].

## 6. FABP4 in the CNS and the Gut–Brain Axis

Several mechanisms link obesity or HFDs to neuroinflammation. First, dysfunction of the BBB associated with obesity permits inflammatory cytokines to infiltrate the CNS [88]. These cytokines activate microglia, which release FABP4 and pro-inflammatory cytokines, thereby exacerbating neuroinflammation. Second, HFDs directly stimulate microglia through elevated fatty acids via the TLR–NF-κB pathway, which enhances the expression of pro-inflammatory cytokines [25,89,90,91,92]. The final pathway suggests a link between the gut microbiota, HFD, and neuroinflammation [93]. Neuroinflammation, an inflammatory process within the CNS, is linked to neurodegenerative diseases such as Alzheimer’s disease, Parkinson’s disease, and motor neuron disease [94].

### 6.1. Microglial FABP4 and Neuroinflammatory Signaling

Microglia play a crucial role in maintaining neuronal–glial circuits by clearing damaged synapses and modulating plasticity [95,96,97]. In cases of obesity, microglia tend to adopt a chronic pro-inflammatory phenotype [91,98]. Persistent elevations in pro-inflammatory molecules, such as IL-1β, TNF-α, reactive oxygen species, and nitric oxide (NO), lead to a reduction in neuronal number and function [99,100].

Recent studies have demonstrated that FABP4 facilitates the release of inflammatory mediators from microglia [35,36]. *FABP4* gene expression was confirmed in both BV-2 and primary mouse microglial cells [101]. So et al. (2022) found that the microglial FABP4-UCP2 (uncoupling protein 2) axis plays a role in regulating neuroinflammation in obese mice [34]. Wild-type mice on an HFD exhibited memory impairment, elevated inflammatory cytokine levels in the hippocampus, microglial activation, and reduced UCP2 expression. In contrast, mice lacking FABP4 showed decreased hippocampal inflammation, increased microglial UCP2 expression, and improved cognitive function. Molecular analysis indicated that FABP4-deficient mice exhibited enhanced metabolic function, reduced oxidative stress, and better energy homeostasis. Wingless/β-catenin signaling is associated with protection against insulin resistance and neuroinflammation [34]. In BV-2 cells, LPS stimulation increased ROS production, pro-inflammatory signaling, JNK phosphorylation, and TLR4 expression, while reducing UCP2 expression; these effects were reversed by FABP4 inhibition [36]. This highlights FABP4’s role in microglial immunometabolism and its potential as a therapeutic target. In both BV-2 and HT-22 cells, HIV1 Tat induced FABP4 expression via NF-κB, creating a feedback loop that resulted in inflammation and neuronal apoptosis. Reducing FABP4 expression inhibits inflammation and neuronal apoptosis in vitro and in a HAND mouse model [102].

### 6.2. Cognitive and Metabolic Consequences of FAB4 Dysregulation

The intricate interactions among diet, the microbiome, and the gut–brain axis have been explored using models ranging from simple organisms to clinical analyses. Research indicates that excessive dietary fat impairs cognitive function in both zebrafish (*Danio rerio*) and laboratory rodents. Meguro et al. (2019) demonstrated that an eight-week HFD diminishes zebrafish’s ability to learn avoidance responses compared to a control diet [103]. Brain transcriptomic analyses have revealed changes in the expression of genes related to neuroplasticity and metabolism as a result of HFDs [103]. In zebrafish, as in mammals, an HFD induces dysbiosis and intestinal inflammation [104]. Enriching zebrafish microbiota with *Lactobacillus rhamnosus* altered the microflora composition and improved fish behavior, including increased exploration and shoal-swimming. Probiotics elevate brain levels of neurotrophic brain-derived neurotrophic factor and serotonin pathway enzymes [104]. This confirms the presence of a gut–brain axis in fish and the influence of microbiota on neurobiology, making zebrafish valuable for mechanistic studies, particularly for observing neuronal development in transparent larvae or generating germ-free forms. Mouse and rat models have demonstrated fundamental relationships: microbiota-deprived mice resist obesity induced by an HFD, and fecal transplants from obese individuals can transfer the obesity phenotype [38]. Vertical microbiota transplantation experiments have shown that gut flora can cause excessive fat accumulation and metabolic disorders, underscoring the critical role of the microbiome in the pathogenesis of obesity and related dysfunctions [38]. In rodents, the direct impact of an HFD on the brain has revealed hypothalamic neuroinflammation; within hours of starting an HFD, microglia and astroglia become activated in the arcuate nucleus of the hypothalamus [105]. This inflammation disrupts the neurons that control appetite, leading to overeating and obesity. Research indicates that microglial FABP4 mediates the brain effects of HFDs; mice lacking microglial FABP4 were protected from cognitive deficits induced by an HFD [34]. HFD mice with normal microglia exhibited impaired memory, increased hippocampal pro-inflammatory cytokine levels, and microglial accumulation [34]. However, FABP4 knockout mice maintained better cognitive function, lower cytokine levels, and reduced microglial proliferation [34]. These mice also showed higher levels of microglial UCP2, suggesting that the absence of FABP4 reduces metabolic stress [34]. FABP4 appears to link microbiota-induced inflammation with neuronal dysfunction and plays a crucial role in the neurodegenerative effects of obesity.

Translational studies in humans have begun to yield preliminary conclusions. They revealed correlations between microbiota composition, intestinal inflammation, and FABP4 levels in obese individuals and those with metabolic syndrome. These patients exhibit reduced diversity in their gut microbiome [33], fewer SCFA producers [38], and an increased Firmicutes/Bacteroidetes (F/B) ratio [33]. This dysbiosis is linked to chronic endotoxemia and low levels of anti-inflammatory defensins in the intestinal mucosa [33,38,61]. These alterations correlate with metabolic parameters, with low butyrate levels linked to increased insulin resistance [62,106]. Circulating FABP4 levels are elevated in individuals with obesity and decline following weight loss and metabolic improvement, supporting its role as a biomarker of metabolic syndrome and cardiovascular disease [32]. Some circulating FABP4 likely originates from macrophages in the visceral adipose tissue, stimulated by bacterial LPS during increased intestinal permeability. The clinical significance of the gut–brain axis in obesity is becoming apparent, with dysbiosis linked to depression and anxiety in obese patients. Modulating the microbiota through a high-fiber diet, probiotics, and fecal transplantation can improve metabolic and cognitive functions. Studies must determine whether microbiome changes cause neurological dysfunction or are a result of them. A feedback loop exists in which obesity and stress affect the microbiota, which in turn exacerbates metabolic and neurohormonal burdens. Understanding these relationships in humans is crucial for developing effective therapies.

**Figure 2:**
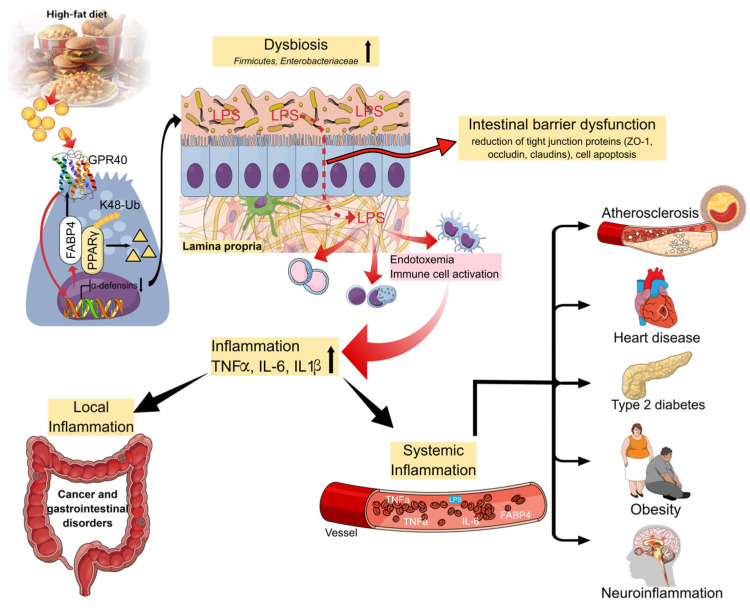
FABP4 as a central mediator linking gut-derived inflammatory signals to systemic and neuroinflammatory outcomes. Intestinal barrier dysfunction under high-fat diet (HFD) conditions promotes the translocation of microbial products, including lipopolysaccharides (LPS), into the circulation. Circulating inflammatory mediators stimulate FABP4 expression and its release from adipocytes, immune cells, and other peripheral tissues. Elevated FABP4 levels amplify inflammatory signaling through NF-κB- and PPARγ-dependent pathways, contributing to chronic systemic inflammation. These processes are associated with the development of metabolic and cardiovascular disorders and facilitate neuroinflammatory signaling via blood-borne cytokines and lipid mediators, ultimately affecting the central nervous system function. In Paneth cells, FABP4 promotes K48-linked ubiquitination and proteasomal degradation of PPARγ, resulting in reduced defensin expression; yellow triangles indicate degradation products of PPARγ. This illustration was created using Mind the Graph (https://www.mindthegraph.com, accessed on 26–28 January 2026).

Research on human intestinal organoids suggests that excess FABP4 reduces defensin production by Paneth cells, potentially fostering the growth of pathogenic bacteria [33]. However, there is a lack of controlled clinical trials that modulate the microbiome while measuring changes in FABP4 and their effects on the brain function. An interventional assessment of the relationship between the microbiota, FABP4, and brain function in humans is required. The systemic consequences of FABP4 activation and its association with neuroinflammation are schematically illustrated in Figure 2.

## 7. Conclusions and Perspectives

Recent evidence has highlighted a clear link between diet, the gut microbiome, and the function of the gut–brain axis. HFD leads to gut dysbiosis, diminishing microbiota diversity and composition, which compromises the gut barrier and permits pro-inflammatory bacterial factors, such as LPS, to enter the circulation [38]. These gut signals trigger chronic inflammation throughout the body and CNS, activating microglial and astroglial cells [105]. This exacerbates neuroinflammation, contributing to the behavioral dysfunctions associated with obesity [5,34]. Several molecules mediate these interactions. SCFAs from healthy microbiota protect the barriers and inhibit inflammation, whereas LPS and excess secondary bile acids from dysbiotic flora initiate inflammatory cascades. The HFD-induced changes also influence FABP4 expression through inflammatory, endocrine, and epithelial pathways. Elevated FABP4 levels act as both local regulators and systemic mediators. In Paneth cells, it suppresses defensins by degrading PPARγ; in epithelial cells and macrophages, it intensifies NF-κB-driven inflammation; and systemically, it circulates as an adipokine, exacerbating metabolic dysfunction. This creates self-reinforcing loops that link the diet, microbiota, barrier function, immune activation, and systemic diseases. These studies suggest that FABP4 functions as an important regulatory mediator, underscoring its significance as both a biomarker and a target for therapy. Modulation of FABP4 through pharmacological inhibition, PPARγ activation, microbiome remodeling, or dietary interventions may represent a potential strategy to preserve defensin expression, support barrier integrity, and mitigate the effects of metabolic diseases. These findings suggest that FABP4 may connect gut dysbiosis to metabolic and neurological disorders. Clinical studies indicate that in obesity, the gut microbiota and FABP4 are associated with inflammation; however, direct interventional trials examining both pathways simultaneously in humans are still needed.

However, knowledge gaps and key research questions persist. Despite these advancements, many facets of the diet, microbiome, and gut–brain axis remain unexplored. The precise molecular mechanisms through which the microbiota influences distant organs, such as the brain, remain unknown. Although primary pathways have been identified, including immunological, vagus nerve, and hormonal pathways, questions remain regarding which specific metabolites and microorganisms predominantly regulate brain function. SCFAs affect microglia via histone deacetylase inhibition and GPRs; however, the dominant pathway in vivo remains unidentified [107]. These SCFAs can have both protective and harmful effects; for example, butyrate inhibits inflammation but may also support immune cell activation as an energy source. The contextual action of microbiota metabolites remains unclear: what determines whether a signal has neuroprotective or neurotoxic effects? Factors such as BBB integrity and vagus nerve function may play a role.

Second, the specific roles of individual cell types in receiving microbiota signals remain unclear. Enteric neurons, gut immune cells, adipocytes, adipose tissue macrophages, and microglia can all respond to signals from the gastrointestinal tract; however, it remains unclear which components are critical for neurological outcomes, as both circulating inflammatory mediators and vagal signaling may contribute in parallel [11]. For example, does neuroinflammation in obesity arise from microglial activation by blood signals (such as LPS and cytokines) or from altered vagal nerve signals originating from the gut? Both mechanisms may operate in tandem. Third, FABP4 has emerged as a potential factor linking the metabolic state with the inflammatory response; however, there is a lack of data on how the microbiota affects the regulation of this protein in various tissues. Studies have indicated that deactivating FABP4 can protect the brain from the effects of HFDs [34]. Could modulating FABP4 levels in the adipose tissue or liver benefit the brain–gut axis, and could FABP4 serve as a therapeutic target for both insulin resistance and obesity-related neuropsychiatric disorders? Finally, most data were derived from animal models that did not fully capture the complexity of humans. The translatability of microbiome-modulating interventions in humans remains uncertain. Large-scale studies are necessary to establish dietary recommendations that support neurological health through beneficial microbiomes.

Researchers have employed advanced experimental strategies to address these gaps. A prominent method involves the use of “omics” techniques for comprehensive large-scale analyses. Metagenomics is used to characterize the composition and metabolic potential of the microbiome, whereas metabolomics profiles metabolites such as SCFAs, bile acids, and bacterially derived neurotransmitters in the blood and cerebrospinal fluid [108]. Additionally, transcriptomics and proteomics of tissues, such as the intestines, brain, and adipose tissue, reveal cellular pathways altered by diet and microbiota signals. These data layers collectively provide a holistic view of the gut–brain axis. In zebrafish studies, RNA sequencing of the forebrain has identified genes significantly affected by HFDs [103]. Future analyses of human tissues may reveal novel signaling molecules.

Advanced in vitro and ex vivo models present alternative approaches. Human microglia or neuron cultures can be co-incubated with serum from HFD animals to examine the direct effects, such as whether serum with endotoxemia induces FABP4 expression in microglia. Intestinal and brain organoids facilitate the recreation of gut–brain axis fragments by cultivating mini-guts with patient microbiota and analyzing metabolites or by developing brain organoids with microglia exposed to these metabolites. Live-cell imaging can reveal the real-time interactions between LPS or SCFAs and neurons.

The significance of germ-free models and microbiota transplantation cannot be overstated. These methods are essential for testing causal hypotheses by transferring microbiota from subjects on an HFD to sterile hosts, allowing researchers to observe the effects of neurological disorders. This process helps to identify the bacterial strains responsible for specific phenotypes. Another strategy involves editing microbiome genes, such as removing bacteria that produce LPS or those that generate specific metabolites, to determine whether this mitigates the negative effects of an HFD on the host.

Currently, in vivo imaging techniques for the gut–brain axis are under development. In animal studies, two-photon microscopy through cranial windows has been used to track microglial activation and BBB permeability following LPS administration. In living humans, radiolabeled ligands for positron emission tomography detect microglial activation, revealing that microglial activity in the hypothalamus correlates with body mass index and inflammation in obese individuals [105]. This imaging technique could be instrumental in assessing whether prebiotic/probiotic interventions can reduce neuroinflammation. New epigenome-editing techniques, such as histone deacetylase inhibitors, are being tested for their ability to modify host responses to the signals of the microbiota. A comprehensive research approach utilizing animal models, advanced in vitro systems, and human in vivo techniques is essential to understand the interactions between diet, the microbiome, and brain health. Future studies should integrate these methods to identify effective therapeutic targets.

It should be noted that obesity disrupts gut–brain communication: gut signals provoke inflammatory responses instead of conveying nutritional status, while the brain’s impaired regulation affects gut barrier function and motility, creating a vicious cycle. Understanding these relationships paves the way for new therapeutic interventions for obesity-related disorders. Merely reducing excess calorie intake is insufficient; the quality of the diet, which influences the microbiome, is crucial. A diet rich in fiber, fermentable oligosaccharides, and polyunsaturated fatty acids fosters beneficial microbiota that produce SCFAs and inhibits LPS translocation [38]. Dietary interventions targeting the microbiome may aid in weight loss and enhance neurocognitive functions. Trials involving probiotics and fecal microbiota transplantation suggest reduced inflammation and improved metabolism in metabolic syndrome [109], although the neurological effects of probiotics require further research. Identifying molecules such as FABP4 in the pathogenesis of obesity complications enables pharmacological interventions. FABP4 inhibitors have improved insulin sensitivity in preclinical models and lipid profiles in mice [32] and may reduce neurological complications by decreasing neuroinflammation. The connections between the microbiota and the brain extend beyond obesity and impact conditions such as depression, Alzheimer’s disease, and multiple sclerosis. Studies on HFDs have provided insights into the impact of the microbiota on the brain. Clinically, patients with obesity require a holistic approach that combines dietary recommendations, physical activity, gut health, and neuropsychological monitoring. Future advancements may lead to the development of predictive microbiome biomarkers and personalized interventions that protect the brain.

While our understanding has grown considerably, future research should focus on several crucial areas. First, long-term clinical studies are essential to evaluate how microbiota modification affects cognitive function, depression risk, and neurodegeneration in overweight individuals. This will facilitate the translation of findings from animal models into clinical guidelines. Second, experimental models should extend beyond laboratory mice to explore the gut–brain axis mechanisms using alternative organisms, such as zebrafish, *Caenorhabditis elegans*, and gnotobiotic models. Third, it is crucial to identify specific bacterial strains with beneficial or detrimental effects, especially those that produce metabolites that reduce FABP4 expression in adipocytes or microglia. These strains may pave the way for targeted probiotics that enhance metabolic and neurological health. Research should also delve into the gut–diet–brain axis concerning sex and age, including the impact of maternal obesity on offspring brain development and sex differences in neuroimmunological responses to HFD consumption. Finally, given the interconnected nature of the human body, the interactions between the gut–brain axis and other systems should be examined. A systemic approach that integrates various axes will offer a more comprehensive understanding of the impact of diet on health.

In conclusion, current evidence supports a model in which an HFD disrupts gut microbial ecosystems and barrier function, initiating inflammatory signaling cascades that extend from the intestine to the peripheral tissues and brain. FABP4 has emerged as an important molecular mediator within this network, integrating dietary lipid exposure, microbiome-derived signals, and inflammatory pathways. Although substantial progress has been made, many mechanistic aspects of microbiome–FABP4–brain communication remain poorly understood, particularly in humans.

Future research should aim to clarify the tissue-specific regulation of FABP4, define the causal relationships between microbial metabolites and host lipid-signaling pathways, and determine the translational relevance of these interactions in clinical settings. A systems-level approach integrating dietary composition, microbial ecology, host metabolism, and neuroimmune signaling is essential for advancing this field.

## 8. Materials and Methods

### 8.1. Literature Search and Data Selection

In May–June 2025, a literature review was conducted using three primary databases: PubMed/MEDLINE, Scopus and Web of Science. To enhance the findings with the most recent reports and open-access studies, Google Scholar was also searched. The queries were crafted by combining the following keywords and their synonyms: “high-fat diet,” “gut microbiota,” “brain-gut axis,” and “intestinal barrier.” Additional terms included: “Firmicutes Bacteroidetes,” “tight junctions,” “zonulin,” “MUC2,” and “MLCK.” These queries were linked using the Boolean operator AND, such as in the following example: (“high-fat diet” AND “gut microbiota” AND “tight junction”). The search was restricted to publications from 2006 to 2025.

Two independent individuals (A.B. and C.D.) conducted title- and abstract-level screenings to assess the relevance of the articles. Studies considered potentially relevant underwent full-text analysis. From each included article, data were extracted on the study model, diet composition (percentage of kcal from fat), microbiota parameters (F/B, alpha- and beta-diversity, selected taxa), intestinal barrier markers (tight junction proteins, zonulin, MUC2, MLCK), microbiome analysis methods (16S rRNA vs. shotgun), and statistical outcomes (mean values, SD/SEM, *p*-value). The collected data were synthesized into comparative tables, and the conclusions were presented in a narrative format.

### 8.2. Inclusion and Exclusion Criteria

#### 8.2.1. Inclusion Criteria

Original articles, including in vivo and in vitro studies, systematic reviews, and meta-analyses, exploring the effects of HFDs on the microbiome, intestinal barrier, or gut–brain axis. These publications were available in English or Polish. The research encompasses animal models, such as mice, rats, and zebrafish, as well as intestinal organoids and clinical studies, both interventional and observational, in humans. The papers are accessible in full text online or through institutional subscription.

#### 8.2.2. Exclusion Criteria

Articles that lack experimental methodology, such as short reports, or those without quantitative data. Studies focusing solely on diets high in simple sugars without differentiating the effects of fats. Very small exploratory pilot studies lacking appropriate control groups were excluded to maintain interpretative robustness. Publications dated before 2010, except for key historical works cited as background information.

### 8.3. Reporting

The PRISMA framework was applied to transparently document the literature selection process within this narrative review and does not imply a full systematic review or meta-analysis. A structured overview of the search process, including the number of records identified and those excluded at the title/abstract and full-text screening stages, is provided in Supplementary Materials (Table S1). The reasons for exclusion were specified at each stage to ensure the transparency and reproducibility of the review.

## Figures and Tables

**Table 1:** Microbiome-derived metabolites regulate FABP4 expression and function.

Metabolite Class	Representative Metabolite(s)	Mechanism of Action	Experimental/Clinical Evidence	Ref.
SCFAs	Propionate	Increases plasma FABP4 and glucagon via sympathetic activation and downstream endocrine effects; links dietary/microbial propionate to systemic FABP4 levels	Mouse and human studies	[8]
	Acetate	Reduces FABP4 expression in neutrophils via SCFA receptor FFAR2; influences apoptosis and lung injury in sepsis models	Preclinical models	[9]
Bile acids	Secondary bile acids	Increased bile acid pools correlate with higher FABP4 mRNA and activation of FXR/PPAR nuclear receptor signaling pathways	Dietary and animal intervention studies	[53]
Oxidized/nitrated lipids	NO_2_-FAs, HODEs	Bind FABP4, activate PPARγ reporter programs, and induce FABP4 expression, establishing a positive amplification loop for PPARγ signaling	In vitro and preclinical studies	[67,68]

FABP4, fatty acid-binding protein 4; FFAR2, free fatty acid receptor 2; FXR, farnesoid X receptor; HODEs, hydroxyoctadecadienoic acids; NO_2_-FAs, nitro fatty acids; PPARγ, peroxisome proliferator-activated receptor γ; SCFAs, short-chain fatty acids.

## Data Availability

No new data were created or analyzed in this study. Data sharing is not applicable to this study.

## Supplementary Materials

The following supporting information can be downloaded at: https://www.mdpi.com/article/10.3390/ijms27052430/s1.

## Abbreviations

The following abbreviations are used in this manuscript:

BBB	Blood–brain barrier
CNS	Central nervous system
ER	Endoplasmic reticulum
F/B	Firmicutes-to-Bacteroidetes ratio
FABP4	Fatty acid-binding protein 4
FFAR2	Free fatty acid receptor 2
FXR	Farnesoid X receptor
GPR	G protein-coupled receptor
HFD	High-fat diet
HODEs	Hydroxyoctadecadienoic acids
IL-1β	Interleukin-1 β
IL-6	Interleukin 6
LPS	Lipopolysaccharide
MLCK	Myosin light chain kinase
MMP-12	Matrix metalloproteinase-12
MUC2	Mucin 2
NF-κB	Nuclear factor kappa-light-chain-enhancer of activated B cells
NO_2_-FAs	Nitro fatty acids
PPARγ	Peroxisome proliferator-activated receptor γ
SCFA	Short-chain fatty acid
TLR	Toll-like receptor
TNF-α	Tumor necrosis factor α
TRAF	TNF receptor-associated factor
UCP2	Uncoupling protein 2
ZO-1	Zonula occludens-1
