# Supplementary material for: The Regulatory Role of FABP4 in Microbiome–Brain–Gut Communication Under High-Fat-Diet Conditions
Source: Int J Mol Sci. 2026 Mar 6;27(5):2430. doi: 10.3390/ijms27052430 (PMC12985547; doi:10.3390/ijms27052430)
Supplement: Supplementary file 1 [file ijms-27-02430-s001.zip › ijms-4173475-supplementary.pdf]

International Journal of
Molecular Sciences

MDPI

Table S1. Summary of the literature search and study selection process according to PRISMA guidelines.

Stage of selection	Database / source	Number of records (n)	Inclusion / exclusion decision
Initial search	PubMed/MEDLINE	28	Included for screening
Initial search			
Initial search	Scopus	17	Included for screening
	Web of Science	9	Included for screening
Initial search	Google Scholar	6	Included for screening
Total records identified			
	–	60	–
Title/abstract screening	All databases	60	Excluded (n = 18)
Title/abstract screening	All databases	42	Included
Full-text assessment	All databases	42	Excluded (n = 12)
Studies included in review	–	30	Included

Disclaimer/Publisher's Note: The statements, opinions and data contained in all publications are solely those of the individual author(s) and contributor(s) and not of MDPI and/or the editor(s). MDPI and/or the editor(s) disclaim responsibility for any injury to people or property resulting from any ideas, methods, instructions or products referred to in the content.
